# A machine-learning parsimonious multivariable predictive model of mortality risk in patients with Covid-19

**DOI:** 10.1038/s41598-021-99905-6

**Published:** 2021-10-27

**Authors:** Rita Murri, Jacopo Lenkowicz, Carlotta Masciocchi, Chiara Iacomini, Massimo Fantoni, Andrea Damiani, Antonio Marchetti, Paolo Domenico Angelo Sergi, Giovanni Arcuri, Alfredo Cesario, Stefano Patarnello, Massimo Antonelli, Rocco Bellantone, Roberto Bernabei, Stefania Boccia, Paolo Calabresi, Andrea Cambieri, Roberto Cauda, Cesare Colosimo, Filippo Crea, Ruggero De Maria, Valerio De Stefano, Francesco Franceschi, Antonio Gasbarrini, Ornella Parolini, Luca Richeldi, Maurizio Sanguinetti, Andrea Urbani, Maurizio Zega, Giovanni Scambia, Vincenzo Valentini, The Gemelli against Covid Group, Alessandro Armuzzi, Marta Barba, Silvia Baroni, Silvia Bellesi, Annarita Bentivoglio, Luigi Marzio Biasucci, Federico Biscetti, Marcello Candelli, Gennaro Capalbo, Paola Cattani, Patrizia Chiusolo, Antonella Cingolani, Giuseppe Corbo, Marcello Covino, Angela Maria Cozzolino, Marilena D’Alfonso, Giulia De Angelis, Gennaro De Pascale, Giovanni Frisullo, Maurizio Gabrielli, Giovanni Gambassi, Matteo Garcovich, Elisa Gremese, Domenico Luca Grieco, Amerigo Iaconelli, Raffaele Iorio, Francesco Landi, Annarita Larici, Giovanna Liuzzo, Riccardo Maviglia, Luca Miele, Massimo Montalto, Luigi Natale, Nicola Nicolotti, Veronica Ojetti, Maurizio Pompili, Brunella Posteraro, Gianni Rapaccini, Riccardo Rinaldi, Elena Rossi, Angelo Santoliquido, Simona Sica, Enrica Tamburrini, Luciana Teofili, Antonia Testa, Alberto Tosoni, Carlo Trani, Francesco Varone, Lorenzo Zileri Dal Verme

**Affiliations:** 1 Sezione di Malattie Infettive, Fondazione Policlinico Universitario A. Gemelli IRCCS, Università Cattolica del Sacro Cuore, Rome, Italy; 2 Fondazione Policlinico Universitario A. Gemelli IRCCS, Rome, Italy; 3 Università Cattolica Sacro Cuore, Rome, Italy

**Keywords:** Machine learning, Risk factors

## Abstract

The COVID-19 pandemic is impressively challenging the healthcare system. Several prognostic models have been validated but few of them are implemented in daily practice. The objective of the study was to validate a machine-learning risk prediction model using easy-to-obtain parameters to help to identify patients with COVID-19 who are at higher risk of death. The training cohort included all patients admitted to Fondazione Policlinico Gemelli with COVID-19 from March 5, 2020, to November 5, 2020. Afterward, the model was tested on all patients admitted to the same hospital with COVID-19 from November 6, 2020, to February 5, 2021. The primary outcome was in-hospital case-fatality risk. The out-of-sample performance of the model was estimated from the training set in terms of Area under the Receiving Operator Curve (AUROC) and classification matrix statistics by averaging the results of fivefold cross validation repeated 3-times and comparing the results with those obtained on the test set. An explanation analysis of the model, based on the SHapley Additive exPlanations (SHAP), is also presented. To assess the subsequent time evolution, the change in paO2/FiO2 (P/F) at 48 h after the baseline measurement was plotted against its baseline value. Among the 921 patients included in the training cohort, 120 died (13%). Variables selected for the model were age, platelet count, SpO2, blood urea nitrogen (BUN), hemoglobin, C-reactive protein, neutrophil count, and sodium. The results of the fivefold cross-validation repeated 3-times gave AUROC of 0.87, and statistics of the classification matrix to the Youden index as follows: sensitivity 0.840, specificity 0.774, negative predictive value 0.971. Then, the model was tested on a new population (n = 1463) in which the case-fatality rate was 22.6%. The test model showed AUROC 0.818, sensitivity 0.813, specificity 0.650, negative predictive value 0.922. Considering the first quartile of the predicted risk score (low-risk score group), the case-fatality rate was 1.6%, 17.8% in the second and third quartile (high-risk score group) and 53.5% in the fourth quartile (very high-risk score group). The three risk score groups showed good discrimination for the P/F value at admission, and a positive correlation was found for the low-risk class to P/F at 48 h after admission (adjusted R-squared = 0.48). We developed a predictive model of death for people with SARS-CoV-2 infection by including only easy-to-obtain variables (abnormal blood count, BUN, C-reactive protein, sodium and lower SpO2). It demonstrated good accuracy and high power of discrimination. The simplicity of the model makes the risk prediction applicable for patients in the Emergency Department, or during hospitalization. Although it is reasonable to assume that the model is also applicable in not-hospitalized persons, only appropriate studies can assess the accuracy of the model also for persons at home.

## Introduction

A rapid spread of SARS-CoV-2, the agent of coronavirus disease 2019 (COVID-19), has been observed first in China since early January 2020 and then in Italy since the last days of February 2020^1^. At this time, the number of COVID-19 cases and related deaths continue to increase. Patients hospitalized with COVID-19 had a relevant rate of clinical deterioration. A first large study on more than 1000 patients with COVID-19 in China reported the need for transfer to an intensive care unit (ICU) in 5% of patients admitted with COVID-19, 2.3% mechanical ventilation; 1.4% died^2^. Other studies have reported a rate around 5% of people admitted as critically ill^3,4^. Case fatality rate in persons with COVID-19 range from < 1 to 15%^5,6^. This implies a staggering challenge for the healthcare system. Unfortunately, treatment options are currently scarce, and as hospital resources are shrinking, systems to target respiratory support and other hospital resources to the highest-risk population, such as the ICU, is a priority. Several predictive models of adverse clinical outcomes in people with COVID-19^7–13^ as well as a systematic review^14^ have been published. Having a clinical algorithm to predict patients who can benefit most from available resources is a valuable aid for decision making and capacity allocation. However, few models have tested the predictive value of simple and readily available parameters. The objective of this study was to generate and validate a machine-learning risk prediction model using parameters that are easily available to help identify patients with COVID-19 who are at higher risk of death.

## Materials and methods

### Study population

The study cohort included all patients admitted to Fondazione Policlinico Gemelli with COVID-19 from March 5, 2020 to February 5, 2021. The diagnosis of SARS-CoV-2 infection was considered when the reverse transcription polymerase chain reaction (PCR) of the SARS-CoV-2 assay was detected from nasopharyngeal swab. For each patient, time 0 was considered the date of hospitalization for SARS-CoV-2 infection.

### Data collection

Patient data included demographics, comorbidities, vital signs, and laboratory characteristics, as well as exposure history, medical history, symptoms at onset, treatment, and outcome data on admission and during hospitalization. Pre-existing conditions collected were diabetes, hypertension, chronic heart disease, chronic respiratory disease, chronic kidney disease, mild to severe liver disease, pancreatitis, neurological impairment, connective tissue disease, transplantation, HIV infection, and malignancy. Vital signs included heart rate, respiratory rate, oxygen saturation by pulse oximetry (SpO2), temperature, body weight, and body mass index (BMI). Laboratory parameters included hematologic variables (white blood cells [WBC], neutrophils, lymphocytes, and eosinophils, platelet count, hematocrit), blood urea nitrogen (BUN); creatinine; total bilirubin; creatine kinase; glucose; sodium; potassium; C-reactive protein; procalcitonine, D-dimer; ferritin; lactate dehydrogenase (LDH); arterial blood oxygen partial pressure (paO2) and inspired oxygen fraction (FiO2), paO2/FiO2 ratio (P/F). SpO2 was grouped into three categories according to the interquartile range: SpO2 less than 94% (first quartile), SpO2 between 94 and 97.0% (second and third quartile), SpO2 greater than 97.0% (fourth quartile). All data were extracted from the electronic medical records of all patients*.* To obtain structural information from unstructured texts (such as clinical diary, radiology reports etc.), Natural Language Processing (NLP) algorithms were applied, based on text mining procedures such as: sentence/word tokenization; rule-based approach supported by annotations defined by the clinical SMEs, and using semantic/syntactic corrections where necessary.

### Outcome

The primary outcome was in-hospital case-fatality rate.

### Predictors

Candidate predictors were included when previously shown to be related to mortality in COVID-19 patients or other respiratory diseases (such as bacterial pneumonia) or possibly related because of clinical plausibility.

### Statistical analysis

To capture the risk of death associated with early hospitalization, we developed a predictive model including only laboratory variables and oxygen saturation at the time of SARS-Cov2 infection. The rationale behind this choice was to provide a tool for early risk assessment. The variables for the model are routinely collected, available within a very short time after presentation, and the literature has reported their association with an increased likelihood of death; moreover, they could also be available at home through home services. In this way, an estimate of risk can be obtained at the time of hospital admission, and actions on the management of critical versus non-critical patients can be readily taken by hospital staff from the patient’s initial clinical status as well as its evolution in a relatively short time frame. A binary logistic regression was applied to express the risk of death in analytical terms, and possibly use it in risk assessment tools based on model coefficients alone. We have chosen to use a logistic regression model because it has both a simple analytical expression and a straightforward interpretation in terms of regression coefficients; other machine learning techniques can have in general higher or slightly higher performances, but at the cost of less technical transferability and clinical explainability, at least in our setting.

Candidate predictors were selected through a combination of prior domain knowledge and a data-driven approach: for example, cut-off values to classify SpO2 and sodium were heuristically defined by the interquartile range, confirmed by a-priori medical knowledge. Overall feature selection was conducted iteratively based on their added contribution to the model in terms of information criterion to minimize model redundancy. The model was trained on the first 8 months of data (March 5, 2020–November 5, 2020), and tested on the next 3 months of data (November 6, 2020–February 5 2021). The out-of-sample performance of the model was estimated from the training set in terms of area under the receiving operator curve (AUROC) and classification matrix statistics by averaging the results of the fivefold cross validation repeated 3-times and comparing the results with those obtained on the test set. Finally, an analysis of lift and gain graphs is presented to identify segments of outcome probability where the model proves particularly useful compared to having no model at all. A model explanation analysis, based on the SHapley Additive exPlanations (SHAP) framework, is also presented to derive information about the contribution of individual variables to the model beyond that obtained from simple logistic regression coefficients.

Baseline laboratory variables for each patient were included by taking the first value after the date-time of hospital admission; only variables with less than 5% of missing values were retained for further analysis, and the final training cohort was selected by choosing the complete records only. This set of variables, along with age and sex, and study outcome, were given as input to a routine of 100-iteration of AIC-based stepwise selection on 80% subsets of the randomly partitioned training data, and characteristics selected at least 50 times were considered to train the final logistic regression model. A level of 0.05 was considered significant for statistical testing. Statistical analysis was done with R version 3.6. Data were stored in SAS Viya V.03.05 and accessed through R with SWAT library version 1.5.0.

According to TRIPOD guidelines^15^, the study should be considered a TRIPOD 2b because it involves a chronological division between training and testing data from a single institution.

### Ethical approval

This study was approved by Ethics Committees of the Fondazione Policlinico Gemelli (IRB 3447). All research was performed in accordance with relevant guidelines/regulations and it was conducted in accordance to the Declaration of Helsinki. Written informed consent was waived because of the rapid emergence of this infectious disease (Comitato Etico Policlinico Gemelli; comitato.etico@policlinicogemelli.it).

## Results

The eligible training cohort included a total of 1126 patients with confirmed COVID-19 admitted from 5 March, 2020, to 5 November, 2020. In this cohort, the in-hospital mortality rate was 13.0%. Characteristics of the study population are shown in Table 1.

**Table 1:** Characteristics of patients included in the training and testing subsets.

Characteristics	Training			Test		
	All patients (N = 921)	Alive (n = 801)	Died (n = 120)	All patients (N = 1463)	Alive (n = 1131)	Died (n = 332)
**Demographics**						
Age, median (SD)	68.0 (15.9)	64.0 (15.4)	84.0 (10.1)	70.0 (18.2)	65.0 (18.5)	80.0 (11.3)
Male	566 (61.4%)	501 (62.5%)	65 (54.2%)	798 (54.5%)	596 (52.7%)	202 (60.8%)
BMI, median (IQR)	26.0 (24.2; 29.1)	26.1 (24.2; 28.7)	26.0 (23.5; 29.3)	26.1 (24.2; 28.2)	26.1 (24.2; 28.2)	26.1 (23.9; 28.1)
**Coexisting conditions**						
Any	685 (74.4%)	574 (71.7%)	111 (92.5%)	1127 (77.0%)	827 (73.1%)	300 (90.4%)
Current or former smoker	24 (2.6%)	23 (2.9%)	1 (0.8%)	23 (1.6%)	20 (1.8%)	3 (0.9%)
Arteriopathy	8 (0.9%)	4 (0.5%)	4 (3.3%)	13 (0.9%)	5 (0.4%)	8 (2.4%)
Chronic liver disease	11 (1.2%)	10 (1.2%)	1 (0.8%)	14 (1.0%)	9 (0.8%)	5 (1.5%)
Cirrhosis	6 (0.7%)	3 (0.4%)	3 (2.5%)	12 (0.8%)	8 (0.7%)	4 (1.2%)
Diabetes	149 (16.2%)	120 (15.0%)	29 (24.2%)	279 (19.1%)	206 (18.2%)	73 (22.0%)
Dyslipidemia	78 (8.5%)	70 (8.7%)	8 (6.7%)	106 (7.2%)	80 (7.1%)	26 (7.8%)
Hiv	27 (2.9%)	26 (3.2%)	1 (0.8%)	22 (1.5%)	18 (1.6%)	4 (1.2%)
Myocardial infarction	116 (12.6%)	87 (10.9%)	29 (24.2%)	227 (15.5%)	148 (13.1%)	79 (23.8%)
Kidney failure	44 (4.8%)	32 (4.0%)	12 (10.0%)	107 (7.3%)	53 (4.7%)	54 (16.3%)
Hypertension	374 (40.6%)	315 (39.3%)	59 (49.2%)	636 (43.5%)	480 (42.4%)	156 (47.0%)
Autoimmune disease	41 (4.5%)	38 (4.7%)	3 (2.5%)	62 (4.2%)	48 (4.2%)	14 (4.2%)
Hematologic neoplasm	6 (0.7%)	4 (0.5%)	2 (1.7%)	29 (2.0%)	16 (1.4%)	13 (3.9%)
Neurologic impairment	102 (11.1%)	59 (7.4%)	43 (35.8%)	171 (11.7%)	101 (8.9%)	70 (21.1%)
Pancreatitis	5 (0.5%)	5 (0.6%)	0 (0.0%)	13 (0.9%)	8 (0.7%)	5 (1.5%)
Cardiovascular pathology	155 (16.8%)	118 (14.7%)	37 (30.8%)	295 (20.2%)	182 (16.1%)	113 (34.0%)
Lung pathology	108 (11.7%)	81 (10.1%)	27 (22.5%)	162 (11.1%)	97 (8.6%)	65 (19.6%)
Radiotherapy	15 (1.6%)	13 (1.6%)	2 (1.7%)	43 (2.9%)	28 (2.5%)	15 (4.5%)
Heart failure	44 (4.8%)	27 (3.4%)	17 (14.2%)	84 (5.7%)	39 (3.4%)	45 (13.6%)
Transplantation	6 (0.7%)	5 (0.6%)	1 (0.8%)	22 (1.5%)	13 (1.1%)	9 (2.7%)
Tumor	236 (25.6%)	188 (23.5%)	48 (40.0%)	544 (37.2%)	406 (35.9%)	138 (41.6%)
Hepatic ulcer	15 (1.6%)	11 (1.4%)	4 (3.3%)	30 (2.1%)	16 (1.4%)	14 (4.2%)
**Symptoms at admission**						
Any	807 (87.6%)	706 (88.1%)	101 (84.2%)	1162 (79.4%)	887 (78.4%)	275 (82.8%)
Cough	344 (37.4%)	327 (40.8%)	17 (14.2%)	366 (25.0%)	309 (27.3%)	57 (17.2%)
Dyspnea	503 (54.6%)	429 (53.6%)	74 (61.7%)	755 (51.6%)	542 (47.9%)	213 (64.2%)
Fever	712 (77.3%)	632 (78.9%)	80 (66.7%)	949 (64.9%)	744 (65.8%)	205 (61.7%)
Nausea or vomiting	47 (5.1%)	43 (5.4%)	4 (3.3%)	65 (4.4%)	53 (4.7%)	12 (3.6%)
Diarrhea	99 (10.7%)	97 (12.1%)	2 (1.7%)	93 (6.4%)	80 (7.1%)	13 (3.9%)
**Time from symptom onset to admission, median (IQR)**	7 (3; 10)	7 (3; 10)	3 (2; 7)	7 (3; 10)	7 (3; 11)	5 (2; 9.5)
**Vital signs on the day of admission, median (IQR)**						
Temperature, °C	37.0 (36.0; 38.3)	37.1 (36.0; 38.4)	36.8 (36.0; 37.9)	36.5 (36.0; 37.9)	36.8 (36; 37.6)	36.3 (36.0; 37.9)
Systolic blood pressure, mmHg	130.0 (118.0; 143.0)	130 (120; 142)	122 (108; 140)	130.0 (118.0; 145.0)	131.0 (120.0; 145.0)	123.5 (110.0; 145.0)
Heart rate, /min	80.0 (71.0; 88.0)	78.0 (70.0; 88.0)	83.0 (72.0; 90.0)	80.0 (73.0; 90.0)	80.0 (74.0; 90.0)	80.0 (72.0; 90.0)
Laboratory findings on the day of admission, median (IQR)						
White blood cell count, /μl	7.0 (5.1; 9.5)	6.9 (5.0; 9.3)	8.1 (5.3; 10.5)	8.1 (5.9; 11.5)	7.9 (6.0; 11.5)	8.5 (5.6; 11.5)
Lymphocyte count, /μl	1.1 (0.8; 1.5)	1.1 (0.8; 1.5)	1.1 (0.7; 1.5)	1.0 (0.7; 1.5)	1.1 (0.8; 1.5)	0.9 (0.6; 1.3)
Hemoglobin level, g/dl	14.3 (13.1; 15.3)	14.5 (13.4; 15.3)	12.9 (11.6; 14.4)	14.1 (12.5; 15.2)	14.3 (12.9; 15.3)	13.3 (11.2; 14.6)
Platelets, μl	198.0 (158.0; 257.0)	202.0 (160.0; 263.0)	176.0 (143.0; 214.0)	204.0 (154.0; 273.0)	207.0 (162.0; 278.0)	196.5 (140.0; 253.5)
Creatinine level, mg/dl	0.9 (0.8; 1.1)	0.9 (0.8; 1.1)	1.1 (0.9; 1.6)	1.0 (0.8; 1.4)	0.9 (0.8; 1.2)	1.3 (0.9; 2.0)
d-Dimer level, ng/ml	740.5 (400.0; 1396.0)	695.0 (380.0; 1314.0)	1158.0 (863.0; 2872.0)	853.0 (468.0; 2031.0)	718.0 (396.0; 1488.0)	1715.5 (811.5; 3667.0)
C-reactive protein level, mg/l	60.4 (23.5; 130.0)	58.0 (22.1; 130.0)	80.4 (38.7; 129.9)	77.4 (32.6; 143.1)	66.4 (25.5; 132.7)	99.9 (61.7; 164.3)
Urea nitrogen, mg/dl	18.0 (15.0; 24.0)	17.0 (14.0; 22.0)	27.0 (20.0; 38.0)	22.0 (17.0; 32.0)	20.0 (16.0; 27.0)	34.0 (23.0; 50.0)
Albumin, g/l	33.0 (30.0; 37.0)	33.0 (30.0; 37.0)	31.0 (26.0; 35.0)	31.0 (28.0; 35.0)	33.0 (29.0; 36.0)	29.0 (26.0; 32.0)
Vitamin D, ng/ml	15.7 (10.7; 20.1)	15.8 (10.7; 20.1)	12.8 (12.8; 12.8)	16.4 (13.2; 28.4)	19.3 (13.2; 28.4)	15.6 (14.3; 22.8)
P/F	290.5 (201.4; 361.9)	297.4 (208.2; 366.7)	248.3 (164.5; 351.7)	228.8 (159.5; 323.0)	249.8 (182.0; 332.8)	166.4 (104.0; 250.0)

*SD* standard deviation, *IQR* interquartile range, *BMI* body mass index, *P/F* paO2/FiO2 ratio.

Survivors differed from nonsurvivors for being younger, having few preexisting medical conditions (specifically, lower rates of diabetes, hypertension, cardiovascular diseases, chronic respiratory diseases, renal failure, solid tumors, and arteriopathy), more cough and diarrhea at onset but less dyspnea, a longer time from symptoms onset to hospitalization, a higher P/F, albumin and hemoglobin value, a higher platelet count, lower WBC and lymphocyte count, a lower creatinine, BUN, C-reactive protein, and D-dimer.

**Figure 1:**
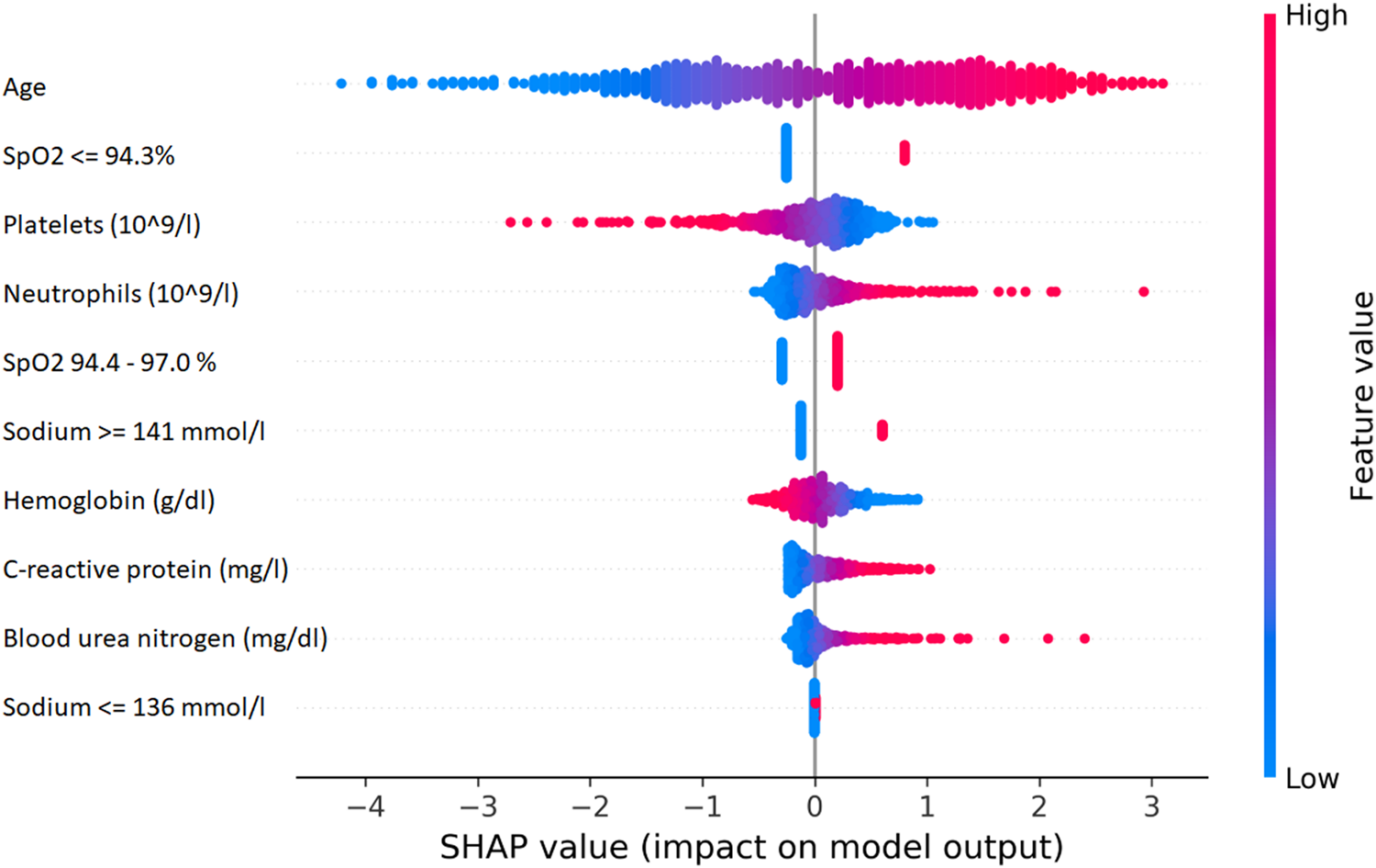
SHAP (SHapley additive exPlanations) framework for the features in the logistic model.

From an initial dataset of 1126 patient records, a total of 921 complete records were included. After the feature selection phase, the selected variables were age (relative selection frequency [RSF] 100%), platelet count (RSF 97%), SpO2 (RSF 80%), BUN (RSF 72%), hemoglobin (RSF 71%), C-reactive protein (RSF 68%), neutrophil count (RSF 60%), and sodium (RSF 58%). These variables were used to fit the logistic regression model. The estimated coefficients of the logistic model are shown in Table 2, along with p values. Each variable in the model is associated with a distribution of importance values among all instances of the dataset (patients), ordered by the value of the variable from low to high. It emerges, for example, that a lower value of platelet count is associated with a higher risk of death, whereas higher values of BUN, C-reactive protein, neutrophils and age are associated with a higher risk of death. The sodium variable was subdivided according to the interquartile range: in this three-category version of the variable (low, normal, high), it can be seen that the “low sodium” group (≤ 136 mmol/l) does not impact death for this cohort of patients, whereas the ”high sodium” class (≥ 141 mmol/l) does. Similarly, SpO2 < 94% has a greater impact in the model than the variable representing SpO2 values between 94 and 97. Figure 1 is a representation of the importance of the variables in the model based on the SHAP framework.

**Table 2:** Logistic regression model at the start of hospitalization for SARS-CoV-2 infection.

Variables	Coefficient	P value
Intercept	− 8.022163	3.34e−09****
Age (continuous)	0.090299	9.32e-15****
Hemoglobin (continous)	− 0.124580	0.03666*
Blood urea nitrogen (continuous)	0.016342	0.00956**
Platelet count (continuous)	− 0.004924	0.00057***
C-reactive protein (continuous)	0.003086	0.04838*
Neutrophils (continuous)	0.092127	0.00203**
Sodium ≤ 136 mmol/l	0.015663	0.95494
Sodium ≥ 141 mmol/l	0.720771	0.01388*
SpO2 94.4–97.0%	0.501530	0.15757
SpO2 ≤ 94.3%	1.060584	0.00521**

**Figure 2:**
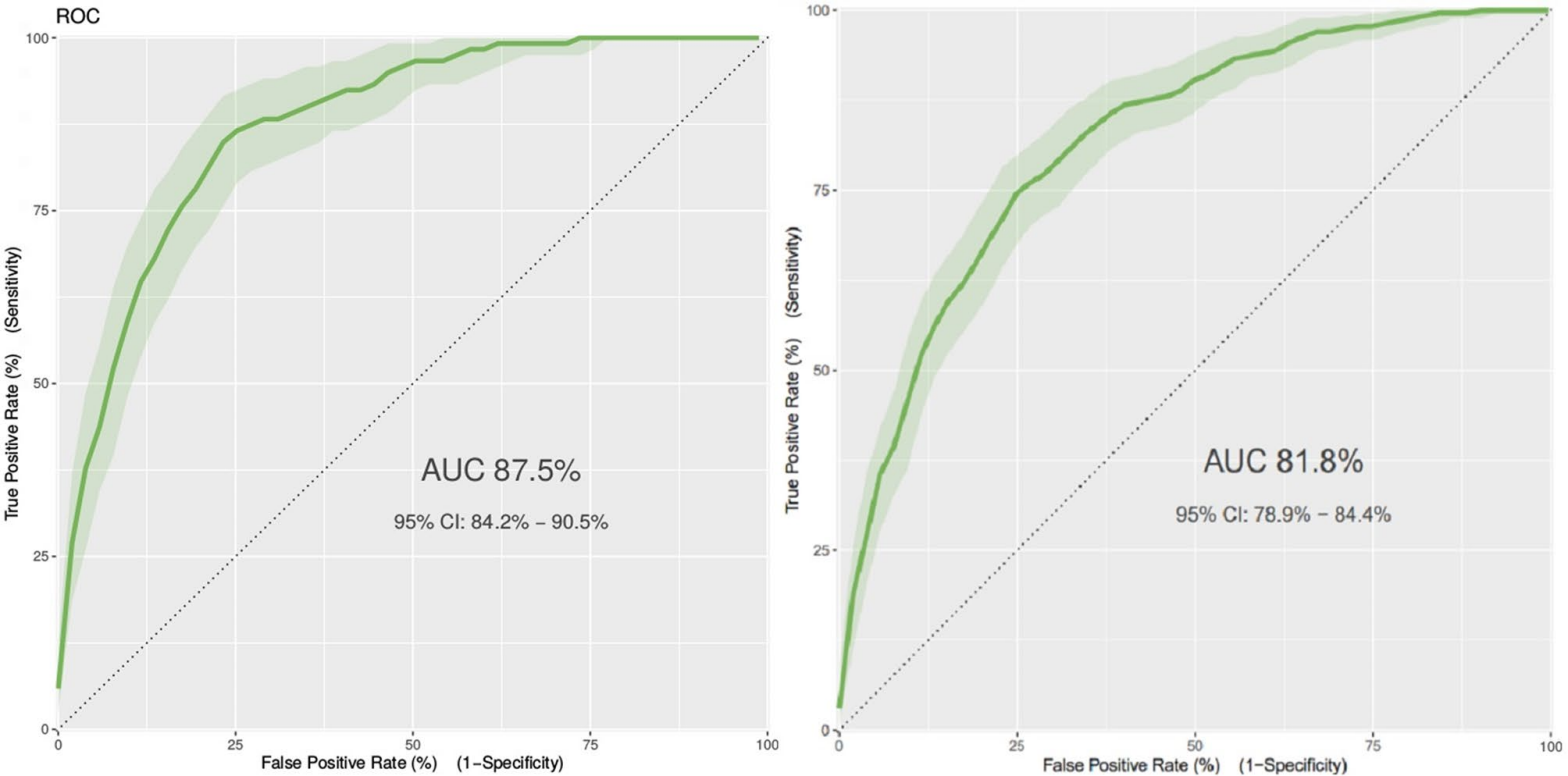
Receiver operator characteristics (ROC) on training set (left) and testing set (right).

**Figure 3:**
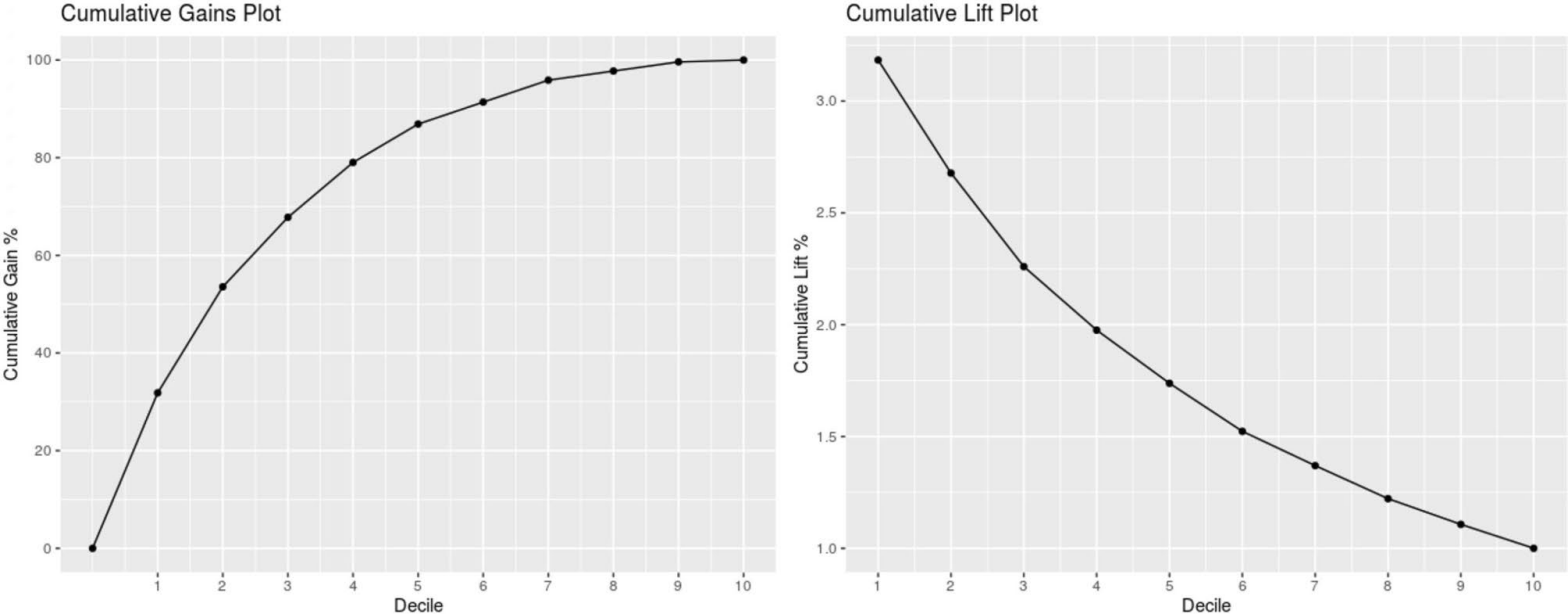
Cumulative gain and lift charts on testing data.

The overall statistical significance of the model according to chi-squared residual deviance test was confirmed with a p-value zero. The fivefold cross-validation repeated 3-times resulted in an AUROC of 0.87, and the statistics of the classification matrix at the Youden index as follows: sensitivity 0.840, specificity 0.774, negative predictive value 0.971. The model was then tested on the cohort of patients admitted between November 6, 2020, and February 5, 2021, (n = 1463), recording the model variable of interest and the clinical outcome. In this cohort of patients, the mortality rate was 22.6%. The model test results in terms of AUROC statistics and confounding matrix are AUROC 0.818, sensitivity 0.813, specificity 0.650, negative predictive value 0.922 (Table 3; Fig. 2). To get a quantification of how the model performs in different segments of probability outputs compared to a random classifier, a gain and lift curve analysis is shown (Fig. 3). Moreover, the lift plot on the testing data in Fig. 3 shows that for the first decile of predictions, the model performs more than 3 times better than random guessing based on prevalence only. Specifically, when considering the first quartile of the predicted risk score on the test set, it contains 6 death events out of 366 total predictions in that risk group. Similarly, the highest 25% of risk scores on the test set contain 196 actual death events, which is more than 50% of the population classified in that risk group (Table 4). A calibration analysis was performed on the testing set to produce the calibration plot of Suppl. Fig. Y. A linear regression fit on the calibration points sampled at every 5 percentiles of the predicted outcome probabilities estimated an intercept of − 4.57 ± 2.12 and a slope of 1.12 ± 0.03 for the regression line with an adjusted R-squared of 0.89. Brier score on the testing set predictions was 0.12.

**Table 3:** Classification matrix and statistics at training set Youden classification threshold on training (cross-validation) and test data.

Dataset	AUROC	Sensitivity	Specificity	PPV	NPV	TN	FN	TP	FP
Training set (cross validation)	0.870	0.840	0.766	0.341	0.971	639	19	100	193
Testing set	0.818	0.813	0.650	0.405	0.922	734	62	270	397

*AUROC* area under the receiver operating characteristics, *PPV* positive predictive value, *NPV* negative predictive value, *TN* true negative, *FN* false negative, *TP* true positive, *FP* false positive.

**Table 4:** Risk groups as defined from gains and lift chart analysis on the test data by applying the thresholds defined on the trained data.

Risk group	Number of patients	Lower threshold	Higher threshold	Death prevalence (%)
Mild risk (< 25th percentile)	366	0.000	0.019	1.6
High risk (25–75th percentile)	731	0.019	0.270	17.8
Very high risk (> 75th percentile)	366	0.270	1.000	53.5

A decision curve analysis was conducted on the testing set to assess model utility compared to baseline strategies of considering “no high-risk” or “all high risk”. Suppl. Fig. W shows the decision curve for thresholds in the range 0–0.5: the curve associated to the model is always higher or substantially higher than the baseline strategies. Also, a zoomed-in version of the graph was produced in Suppl. Fig. Z to highlight the first risk threshold we identified (0.02) for the risk classes.

### Evolution of respiratory condition by initial risk group

**Figure 4:**
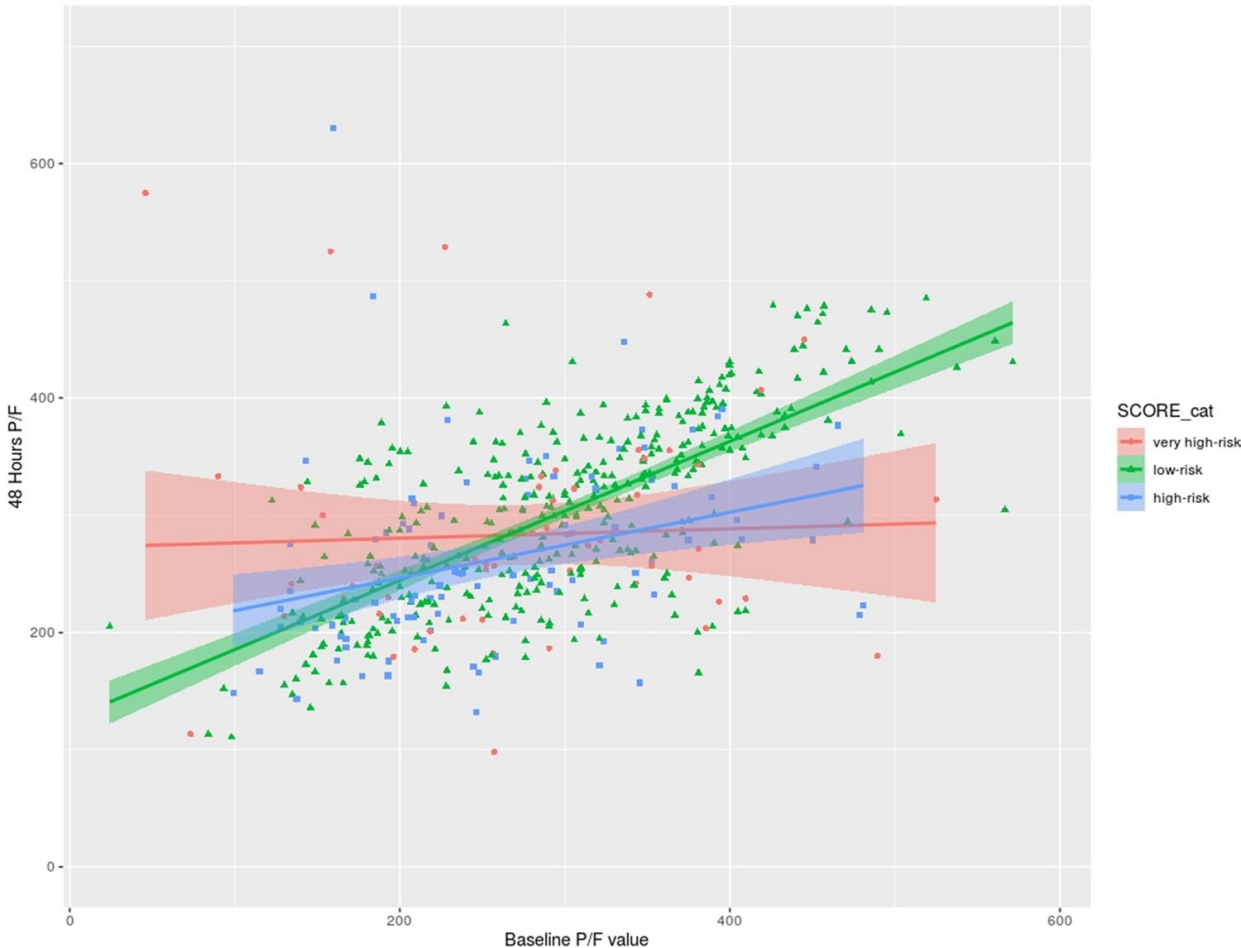
Scatter plot of baseline P/F value and its variation at 48 h for the three groups of risk class patients according to the logistic regression model. The line of best linear fit is reported for ease of visualization.

In addition to having an instrument capable of distinguish between low-risk, high-risk and very high-risk cases with a fair degree of accuracy, we evaluated the evolution of the different groups of patients in the first few hours after hospital admission. Considering the cohort of patients used for model training and taking the first available value of P/F within 24 h of hospital admission, the three model-defined risk groups had a mean value of P/F of 301, 273, 273 for low-risk, high-risk and very high-risk, respectively. A t test between the low-risk group versus the other two categories showed a statistically significant difference. To assess the subsequent time course, the change in P/F at 48 h after the baseline measurement can be plotted against its baseline value (Fig. 4). In the low-risk group, the P/F following the admission to hospital did not worsen over the following 48 h (adjusted R squared of 0.48). In the very high-risk group the P/F tends to a single value independently from the baseline value (adjusted R squared of 0).

### Adoption in clinical practice

**Figure 5:**
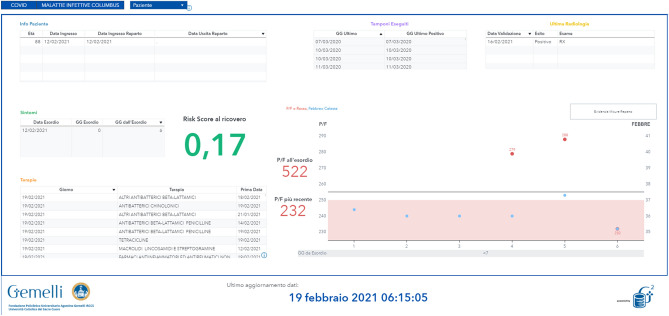
Example of the availability on HER of the risk score of death at admission for a patient with SARS-CoV-2 infection.

The risk of death score for each patient with SARS-CoV-2 infection was made available to clinicians along with real-time predictions directly on the Electronic Health Record (Fig. 5).

## Discussion

Given the high rate of patients with complications of SARS-CoV-2 infection, prioritization of patients who need higher levels of care or immediate medical attention is critical. In the present study on a total of 2384 patients hospitalized with COVID-19, of whom 18.9% died, we presented an artificial intelligence-driven clinical algorithm to predict risk of death. The algorithm showed that abnormal blood counts (hemoglobin, platelets, neutrophils), high levels of BUN, C-reactive protein, sodium and lower SpO2 were associated with an increased risk of death. From the model, we were able to identify three risk level groups: *low-risk*, with a prevalence of death of 1.6%, *high-risk*, with a prevalence of death of 17.8%, and *very high-risk* with a prevalence of death of 53.5%. Our model includes only easy-to-obtain variables: its simplicity makes the risk prediction applicable for different purposes for patients in the Emergency Department, or during the hospitalization. For example, when the calculated individual risk of death is low, the physician may choose to monitor the patient and send him/her back home, whereas high risk estimates suggest more aggressive monitoring or resource allocation or may be useful in anticipating organizational needs in terms of intensive, sub-intensive, and rehabilitation rooms and staff allocation*.* Safely discharging patients from the Emergency Department is of a great benefit in saving beds for other critically ill patients. Such a parsimonious model is exploitable even in medically resource-limited settings.

The discriminatory performance of the model is very high and testing of the model on a new cohort of the very newly diagnosed patients confirmed its validation. The model also demonstrated good accuracy in predicting respiratory evolution when P/F at baseline and at 48 h were considered.

The two major strengths of the present study are the parsimonious inclusion of simple and easy-to-obtain variables, also available in primary care settings, and the immediate translation of a mathematical model into a comprehensible and implementable number in EHR for clinical decision making in daily practice.

Several published studies provide a computational tool or Web-based calculator for easy use in a variety of settings^10,11,16–20^. Unfortunately, such calculators require data entry that is cumbersome in a busy clinical practice. Real-time processing of the model directly from the EHR provides an immediate and seamless calculation, a score that can be used to support clinical decision making and support prioritization, especially when the healthcare system is overloaded.

Other predictive models have been published previously, many of which report age, hematologic measures, C-reactive protein and spO2 as the main variables explaining the predictive model^7,8^. Most of the published studies focused on very critically ill people^21^. Our results confirm and extend those of other large cohort studies^7–13^ demonstrating the predictive value of renal function^20,22,23^ and, particularly, of blood urea nitrogen for mortality^14,24,25^. In addition, we share 4 of 9 variables from a machine-learning-based study with the largest included population^14^. Many models make particular use of easy-to-collect variables^26,27^.

The model of the present study shares some variables among those included in CURB-65, a well-validated and widely used score for predicting mortality in persons with community-acquired pneumonia^28^, with an AUROC of 0.72 (0.71–0.73) in patients with COVID-19^14^. Age and BUN are included in both CURB-65 and our predictive model. whereas respiratory function was described by respiratory rate in CURB-65 and SpO2 in our model. The variables in the present model also share many parameters with other risk scores used to predict mortality in patients with sepsis, such as the widely used SOFA score^29^, probably reflecting a clinical presentation of COVID-19 very close to sepsis. These findings may help highlight the complex pathogenesis of the SARS-CoV-2 infection.

To date, published models implementing machine learning techniques for statistical analysis used very different techniques (support vector machine^27^, artificial neural networks, decision trees, partial least squares discriminant analysis, K nearest neighbour algorithm^22,30,31^, ensemble, Gaussian process, linear, Naïve Bayes^22^, random forest, catboost, and extreme gradient boosting^31^) indicating good ability to predict mortality. In our study, we proposed a simple classifier model based on logistic regression which can be easily exported on different software environments and has a neat clinical explainability in terms of regression coefficients, while still maintain a satisfying out-of-sample performance. In addition, we enhanced even more the model readability by using the Shapley additive explanations (SHAP) framework to make the individual variables contribution to the overall prediction available and understandable in real-time to physicians along with the model’s risk score. Machine learning methods can synthesize data from thousands of patients to generate tailored predictions for each new patient in real time. In addition, model explanations used in our study such as Shapley additive explanations (SHAP)^25,27^ were made available and understandable to physicians along with real-time predictions.

The present study includes several limitations: the scalability and the interoperability of the entire data architecture must be demonstrated in other centers and clinical settings. Moreover, the impact of clinical implementation of this predictive model in daily clinical life has not yet been demonstrated. Studies demonstrating changes in clinical management based on model prediction are strongly warranted.

The two greatest strengths of the present study are the parsimonious inclusion of simple and easy-to-obtain variables, also available in primary care settings, and the immediate translation of a mathematical model into a comprehensible and implementable number in EHR for clinical decision-making in daily practice. Indeed, for each patient who tested positive to PCR for SARS-CoV2, hospital IT made available to us in near real-time the patient’s data in a pseudo-anonimyzed manner on a dedicated environment. We were able to access this data and send back to the server the model risk score, the risk class, and the importance of the variables for each particular prediction. This output information was entered into the EMR software interface of the emergency and infectious disease, through an automated procedure, for on-line consultation in the wards.

Currently, containing the COVID-19 epidemic is an urgent global priority. Dealing with a severe pandemic disease such as COVID-19 is also very challenging because rapidly changing variables (vaccination, new SARS-CoV-2 variants, saturation of hospital capacity) alter the risk of death over time^32^. Our predictive model is pragmatic and effective in identifying individuals at particularly high risk for a poorer hospital course. Computational infrastructure could enhance this process, and data repository, updated in real time, can continuously inform the planning of diagnostic and treatment strategies. Future randomised trials should be conducted to demonstrate whether the current use of the death risk score will improve final patient outcomes. Predictive models can help provide appropriate care and optimize the use of limited resources, such as during a pandemic.

Finally, sharing large amounts of data among centers around the world can be a formidable response to the tremendous challenge of the COVID-19 pandemic.

## Supplementary Information

Supplementary Information.
