# Supplementary material for: A machine-learning parsimonious multivariable predictive model of mortality risk in patients with Covid-19
Source: Sci Rep. 2021 Oct 27;11:21136. doi: 10.1038/s41598-021-99905-6 (PMC8551240; doi:10.1038/s41598-021-99905-6)

Suppl. Fig. Y Model's calibration plot on the testing set

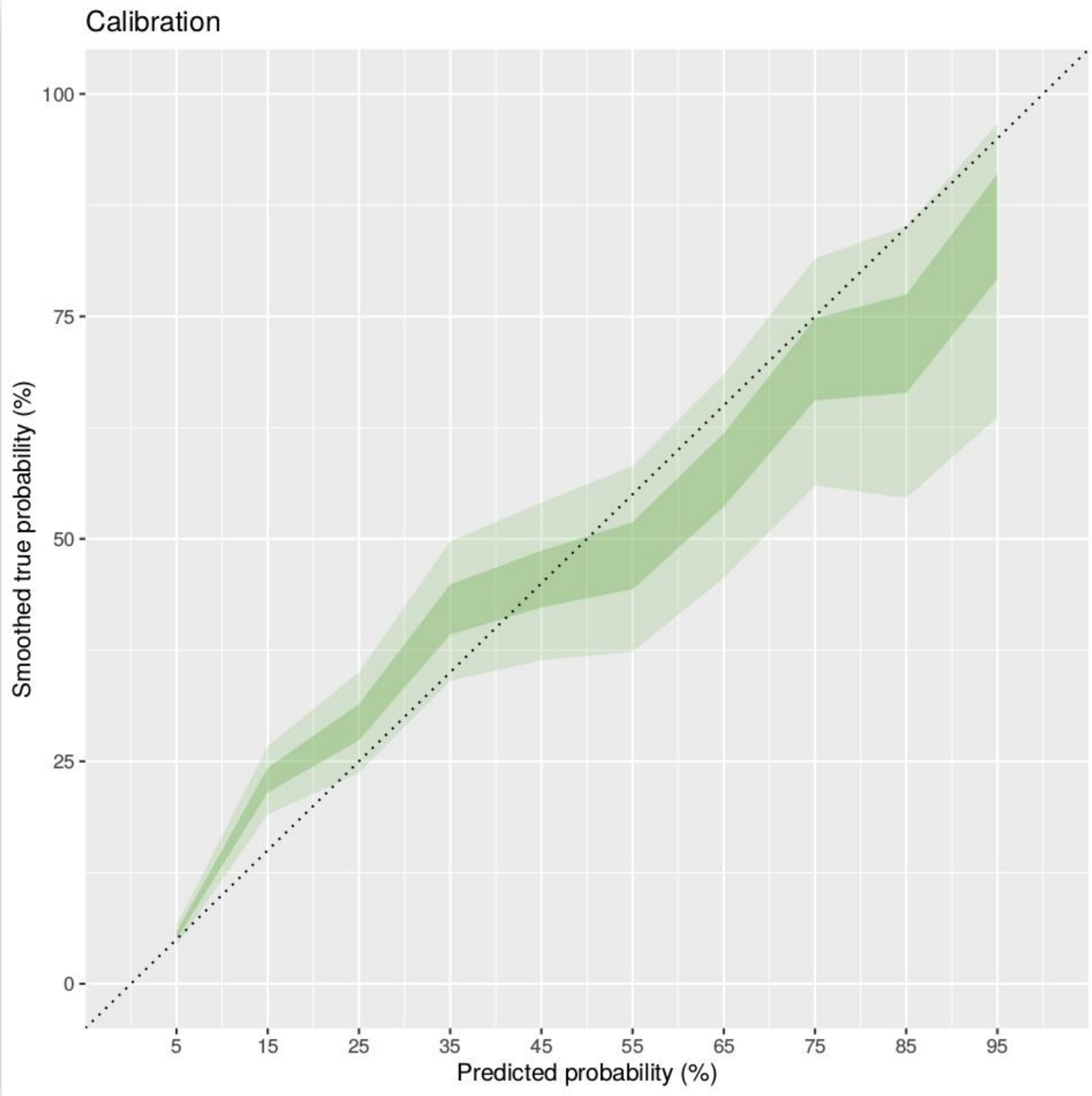

Suppl. Fig. W. Decision curve analysis for the risk threshold in the range 0 to 0.5

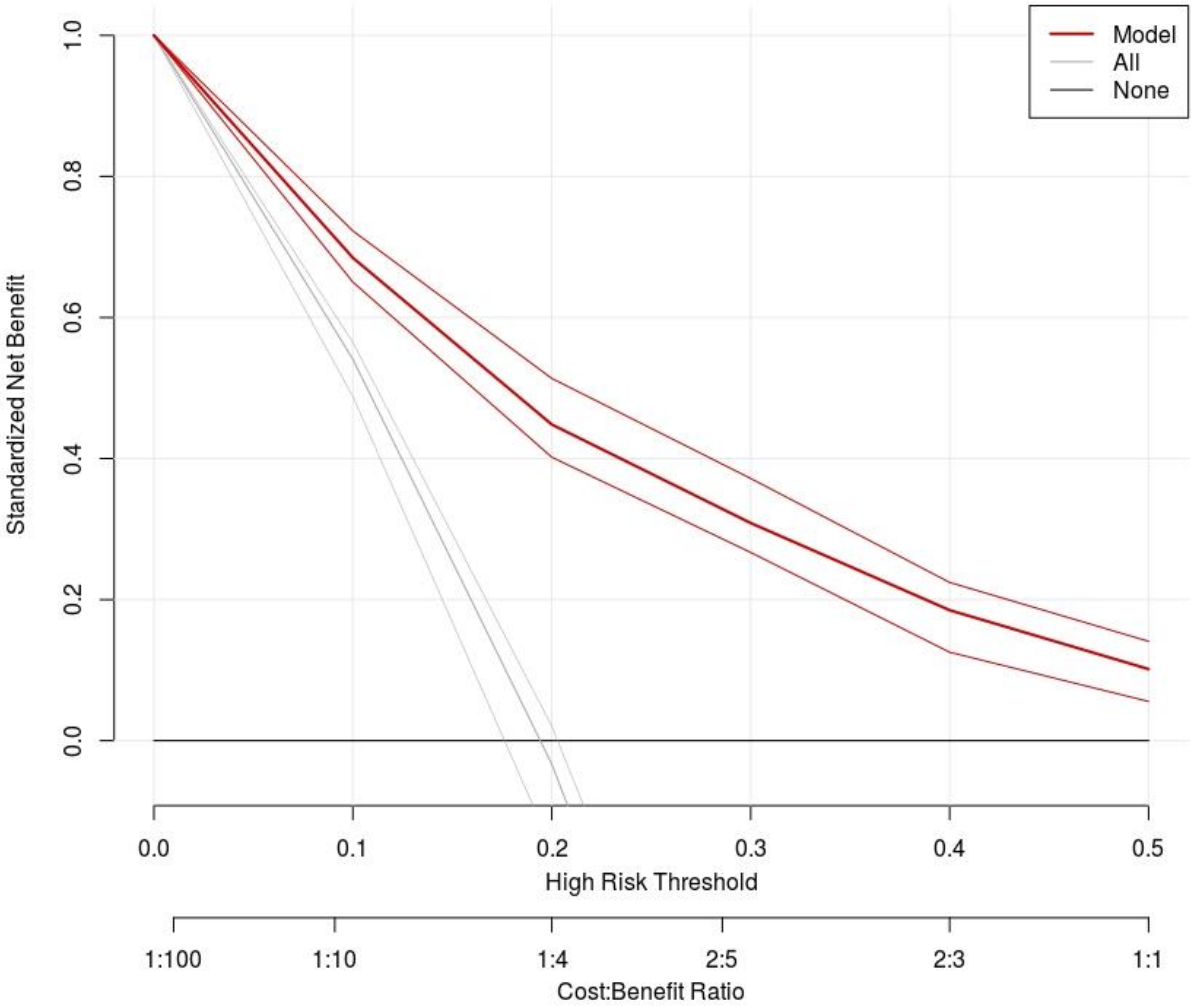

Suppl Fig Z A zoomed-in version of the Fig W to highlight the first risk threshold we identified (0.02) for the risk classes

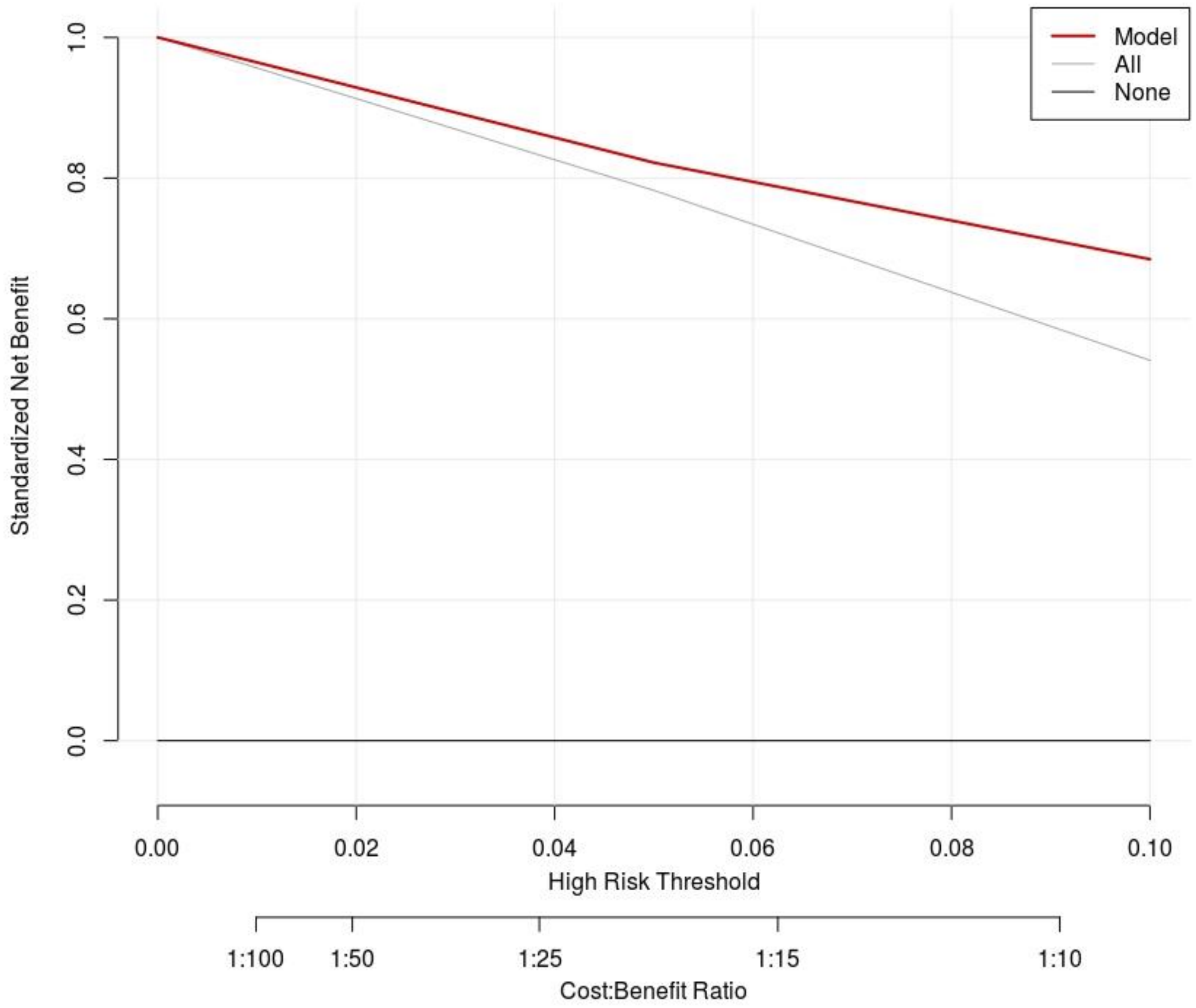

Supplement: Supplementary file 1 — Supplementary Information. [file 41598_2021_99905_MOESM1_ESM.pdf]
